# Quantitative Analysis of Torso FDG-PET Scans by Using Anatomical Standardization of Normal Cases from Thorough Physical Examinations

**DOI:** 10.1371/journal.pone.0125713

**Published:** 2015-05-28

**Authors:** Takeshi Hara, Tatsunori Kobayashi, Satoshi Ito, Xiangrong Zhou, Tetsuro Katafuchi, Hiroshi Fujita

**Affiliations:** 1 Department of Intelligent Image Information, Division of Regeneration and Advanced Medical Sciences, Gifu University Graduate School of Medicine, Gifu, Gifu, Japan; 2 Department of Radiological Science, Faculty of Health Sciences, Junshin Gakuen University, Fukuoka, Fukuoka, Japan; 3 Department of Radiology, Daiyukai General Hospital, Ichinomiya, Aichi, Japan; 4 Department of Radiological Technology, Gifu University of Medical Science, Seki, Gifu, Japan; University of Cambridge, UNITED KINGDOM

## Abstract

Understanding of standardized uptake value (SUV) of 2-deoxy-2-[18F]fluoro-d-glucose positron emission tomography (FDG-PET) depends on the background accumulations of glucose because the SUV often varies the status of patients. The purpose of this study was to develop a new method for quantitative analysis of SUV of FDG-PET scan images. The method included an anatomical standardization and a statistical comparison with normal cases by using Z-score that are often used in SPM or 3D-SSP approach for brain function analysis. Our scheme consisted of two approaches, which included the construction of a normal model and the determination of the SUV scores as Z-score index for measuring the abnormality of an FDG-PET scan image. To construct the normal torso model, all of the normal images were registered into one shape, which indicated the normal range of SUV at all voxels. The image deformation process consisted of a whole body rigid registration of shoulder to bladder region and liver registration and a non-linear registration of body surface by using the thin-plate spline technique. In order to validate usefulness of our method, we segment suspicious regions on FDG-PET images manually, and obtained the Z-scores of the regions based on the corresponding voxels that stores the mean and the standard deviations from the normal model. We collected 243 (143 males and 100 females) normal cases to construct the normal model. We also extracted 432 abnormal spots from 63 abnormal cases (73 cancer lesions) to validate the Z-scores. The Z-scores of 417 out of 432 abnormal spots were higher than 2.0, which statistically indicated the severity of the spots. In conclusions, the Z-scores obtained by our computerized scheme with anatomical standardization of torso region would be useful for visualization and detection of subtle lesions on FDG-PET scan images even when the SUV may not clearly show an abnormality.

## Background

Positron emission tomography (PET) with 2-deoxy-2-[18F]fluoro-d-glucose (FDG) scanning is an excellent diagnostic method for detecting cancers because the lesions utilize higher glucose than normal regions. Standardized uptake value (SUV), which is defined as the regional tissue radioactivity concentration normalized for the injected dose and body surface area, is a semi-quantitative measurement of FDG metabolism[1, 2]. However, the problem with the SUV is that it is subjected to many sources of variability[3, 4]. In general, the SUV in normal tissues or organs can be estimated as a relatively low value, such as 2.0. However, if a confidence interval for each normal organ can be determined, the interval may improve the accuracy of interpretation and familiarity with the normal patterns in FDG.

The concept of comparing the abnormal and normal groups has been proposed as a statistical analysis technique. Researchers have proposed statistical analysis of functional images for diagnosis of dementia and other brain functions[5–9], whole-body bone scans[10, 11]; however, a statistical approach for analyzing torso FDG-PET images has not been proposed yet. Some researchers have recognized the importance of determining the confidence intervals of SUVs from normal organs. Engel et al. reported the normal activity accumulations in whole-body FDG-PET scan images[12]. They concluded that the diagnostician must be familiar with the normal accumulations of FDG. Wang et al. also reported the characteristics of physiological FDG for normal tissues, and the result showed that the mean SUVs in normal tissues were sometimes higher than 2.5[13]. This result implies that an understanding of the SUV range for normal tissue is valuable for improving the diagnostic accuracy. They also concluded that the automated comparison of the SUVs from patients with normal SUVs in the database might be useful.

The most difficult things for the statistical image analysis are the constructing the normal data as control cases. Normal brain developments for quantitative analysis for the function in healthy volunteers have to be done before the brain function analysis using MR or PET scans. In Japan, thorough medical examinations for healthy people by using FDG-PET scan are available for the early detection of cancer in torso region[14–19].

We have studied the fundamental technique of image deformation for the torso region in FDG-PET scan images and have reported the technical aspects in some conferences, but the evaluation of our scheme by using many abnormal accumulations has not been reported.

## Purpose

This study aimed to develop a new method for quantitative analysis of SUV of torso FDG-PET scan images to examine for abnormality by use of Z-score, which is based on a range of SUVs in normal cases by using an anatomical standardization approach and to show the usefulness of Z-score by comparing accumulations of normal and subtle abnormal regions.

## Materials and Methods

### Ethics

The IRB in Gifu University approved this study (#23–131). This study was designed as retrospective study with written informed consent for the notion of general consent from the patients before the examination. The linkable anonymizing was applied to the data conversion from clinical departments to our lab. The code book was stored at only the clinical departments.

### Database

Two hundred forty three (male: 143, female: 100) normal and 63 abnormal cases were collected from a hospital database. A board-certificated nuclear medicine physician interpreted all of the cases. The detail of normal patients’ cases to determine the normal range are summarized in Table 1. The normal data were collected from cancer screening using FDG-PET, which was employed for advanced medical examination of healthy people in Japan. We retrospectively collected the normal and the abnormal image data based on the patient records.

**Table 1:** Characteristics of the normal patients.

	Height(cm)	Weight(kg)	Age
**Male (n = 143)**	169 [153, 184]	70 [47, 110]	55 [34, 84]
**Female (n = 100)**	154 [142, 163]	51 [33, 80]	55 [39, 69]

mean [min, max]

The normal cases in this study met the following five criteria:

#1 No patient record of medical treatment within one year after the FDG-PET examination.
#2 No subjective judgment of existing any abnormal spots by a board-certified radiologist for nuclear medicine.
#3 No subjective judgment of existing any abnormal spots by two radiological technologists.
#4 No obvious renal failure
#5 Body Mass Index (BMI) is less than 36. The abnormal group may have abnormalities in more than one organ. A radiological technologist segmented 432 abnormal accumulations from 63 abnormal cases. The physician and another technologist verified the location of the abnormal accumulations on PET scans. All of the abnormal spots were from cancer cases with biopsy proven. The abnormal cases were identified on the basis of follow-up PET examinations. A single intravenous injection (140–200 MBq) of FDG was administered via an upper-extremity vein. The patients underwent PET imaging (Discovery LS; GE Medical Systems) after 1 h of post-injection rest, and they were instructed to breathe shallowly during the scan to minimize image blurring. To restrain muscle activity, the imaging protocol of FDG-PET scan was followed by the guideline [20]. The activity of normal brown fat was considered as normal variation in the normal database.

### Image registration

A novel image registration approach, which we developed, was employed for the automated extraction of various organs in the torso region. This approach facilitated the calculation of the mean and the standard deviation (SD) of SUV from normal cases. All of the normal cases were deformed into a predefined standard torso configuration as follows. First, the body region was extracted by thresholding the SUV at 0.5 and by using a labeling technique. After labeling, the region with the maximum volume was selected. This was to eliminate noise and other high accumulations outside the body. Because the SUV of a region outside the body is usually very low, an appropriate threshold value can be used to exclude the outside region. After this preprocessing, the torso region was registered with the standard torso configuration. The registration approach consisted of the following 3 automated steps:

1. Physique registration
2. Organ registration
3. Surface registration.

**Fig 1:**
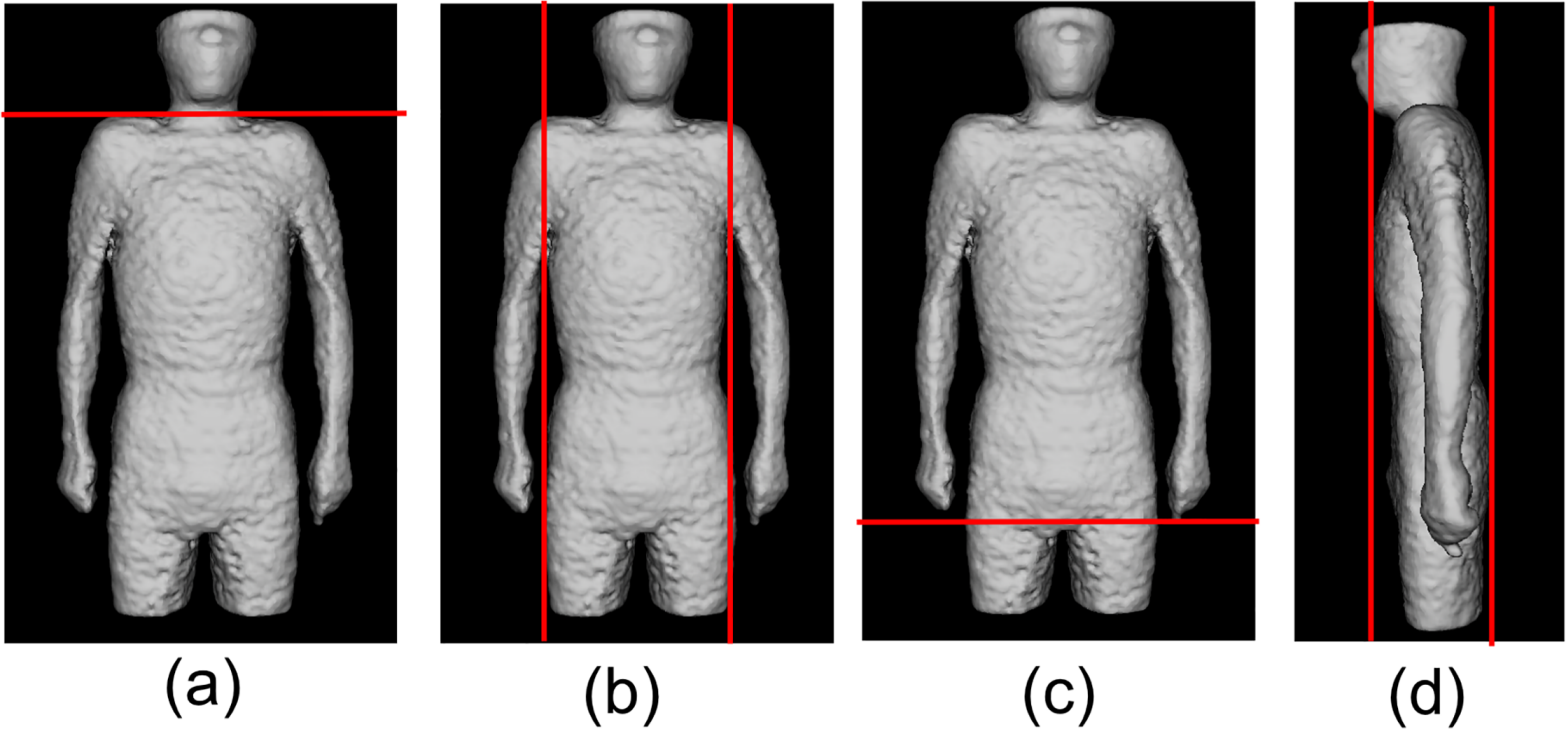
Determined planes (red lines) to extract torso region. (a) Neck-Shoulder (NS) plane. (b) Arms-Chest (AC) planes (left/right). (c) Thigh-Hips (TH) plane. (d) Anterior and Posterior (A, P) planes.

**Fig 2:**
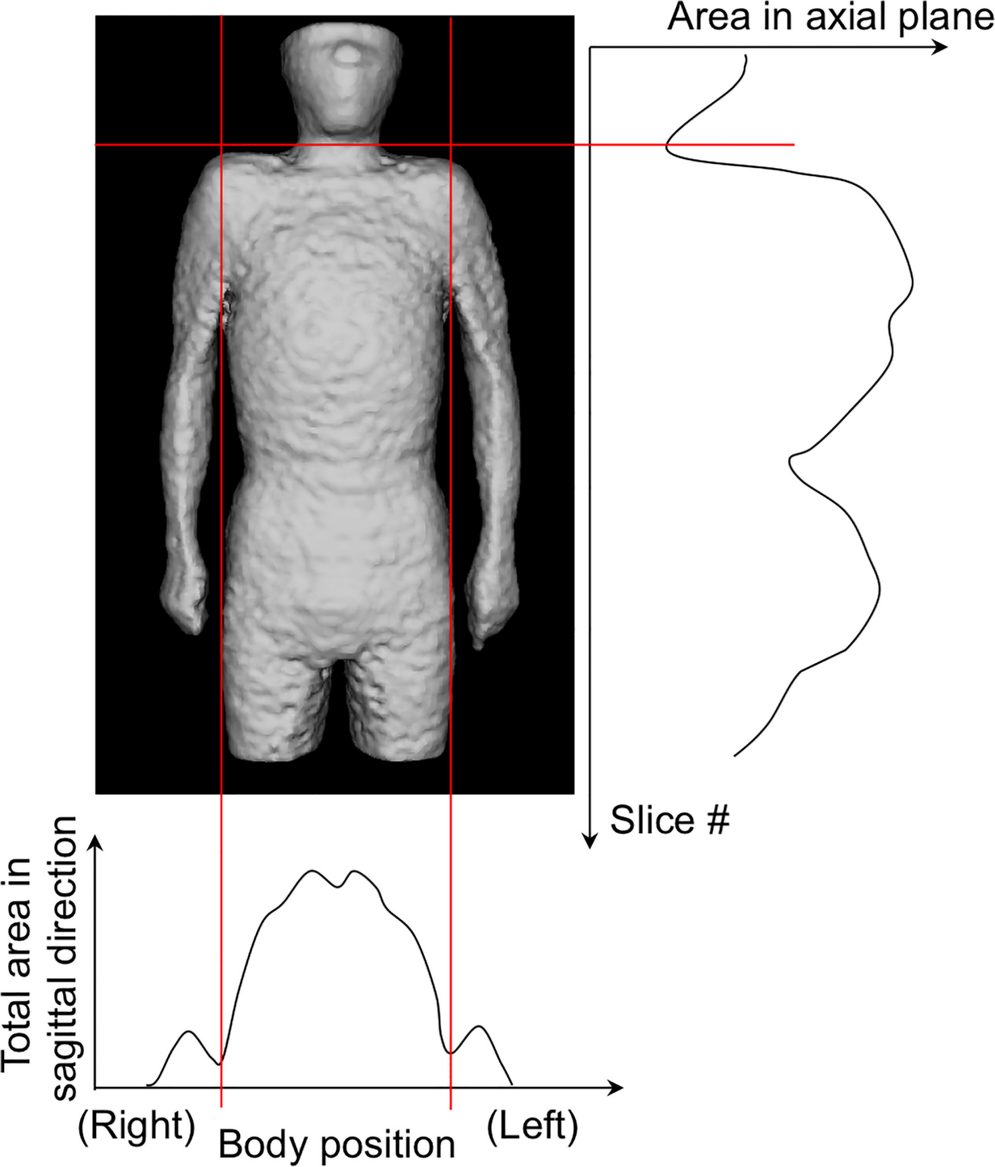
Area distributions in sagittal and axial directions to determine NS and AC planes.

**Fig 3:**
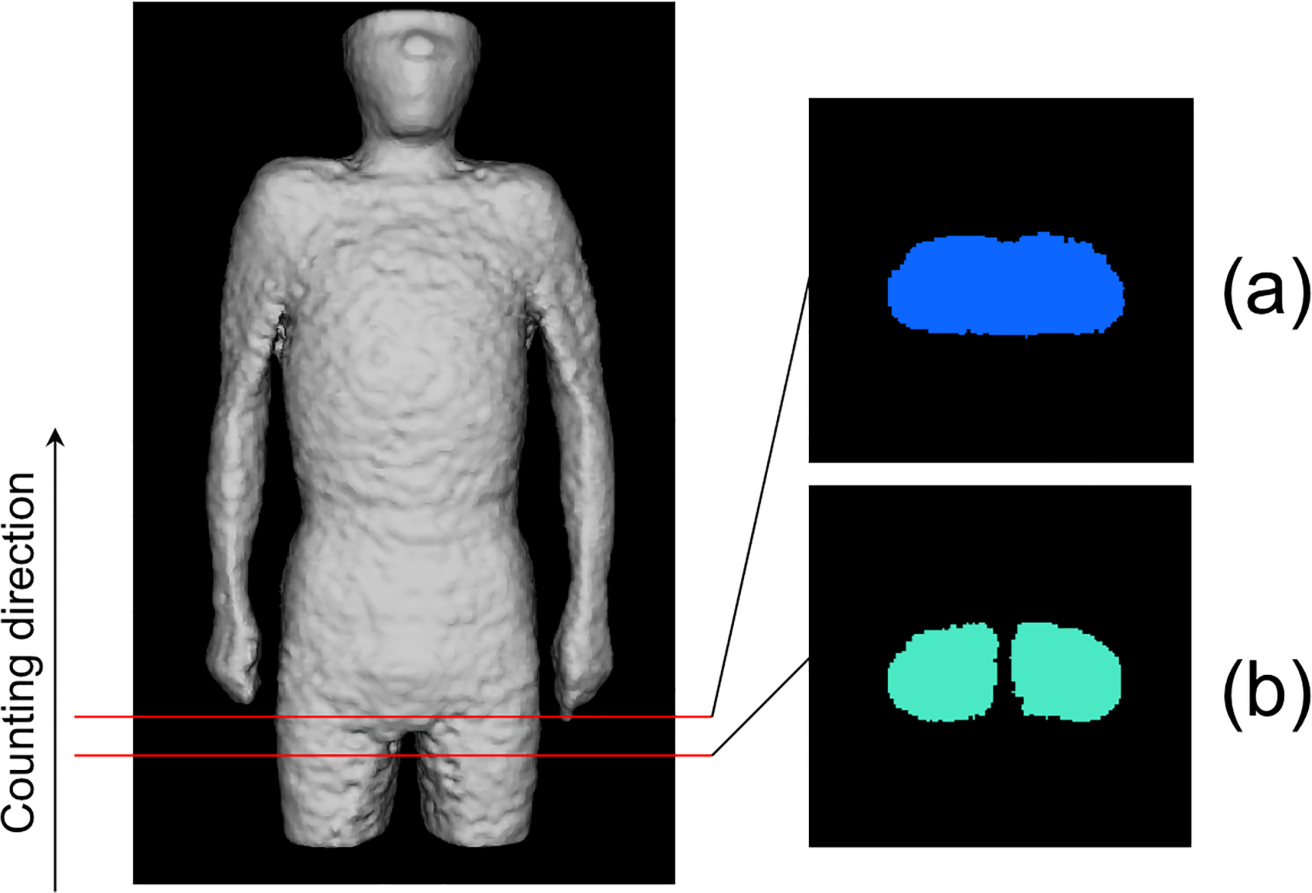
An example of labeled images in waist (a) and thigh (b) regions to be used for determining the TH plane.

**Fig 4:**
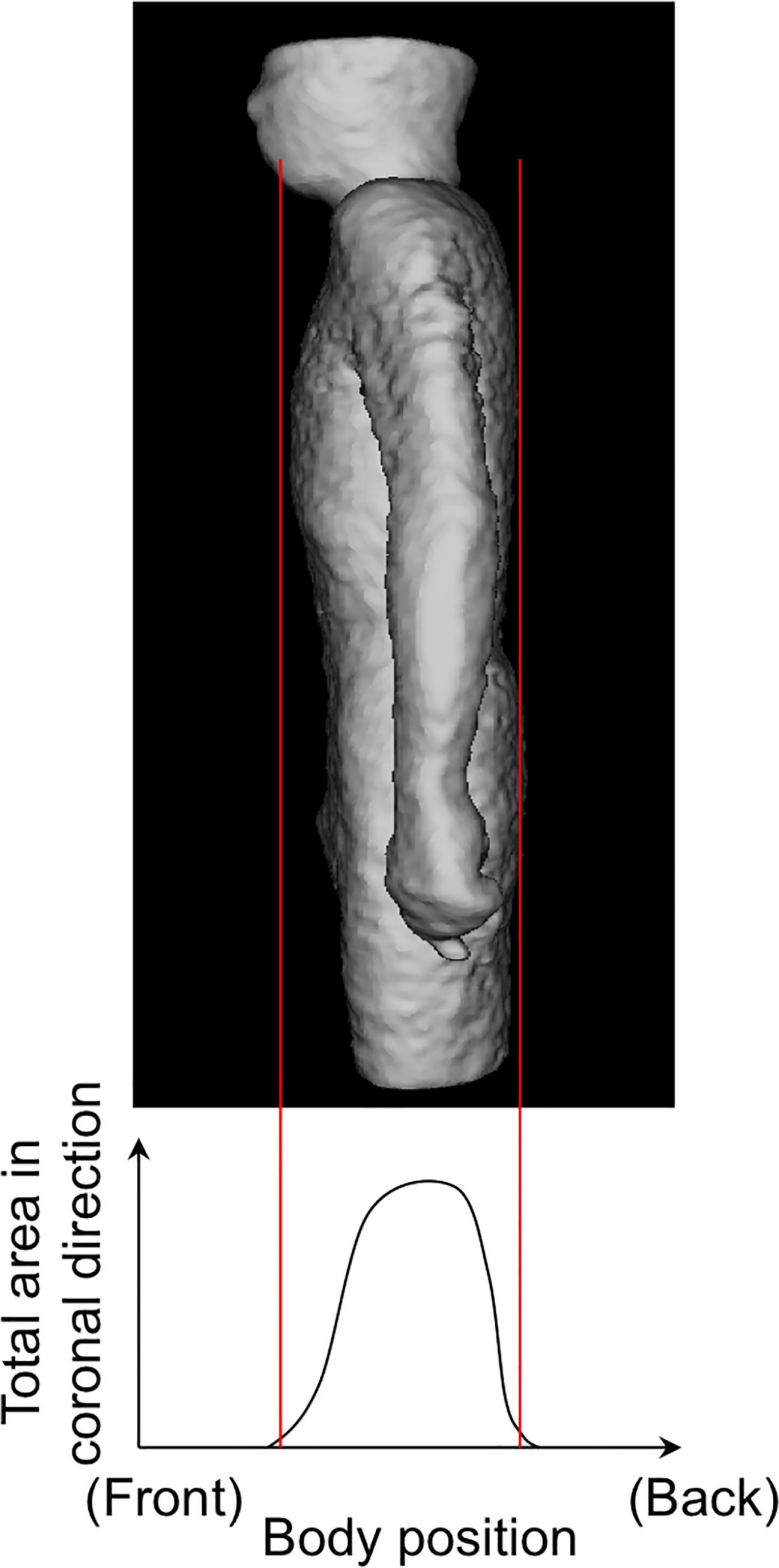
Area distributions in coronal direction to determine Anterior (A) and posterior (P) planes. Head region was excluded to count.

**Fig 5:**
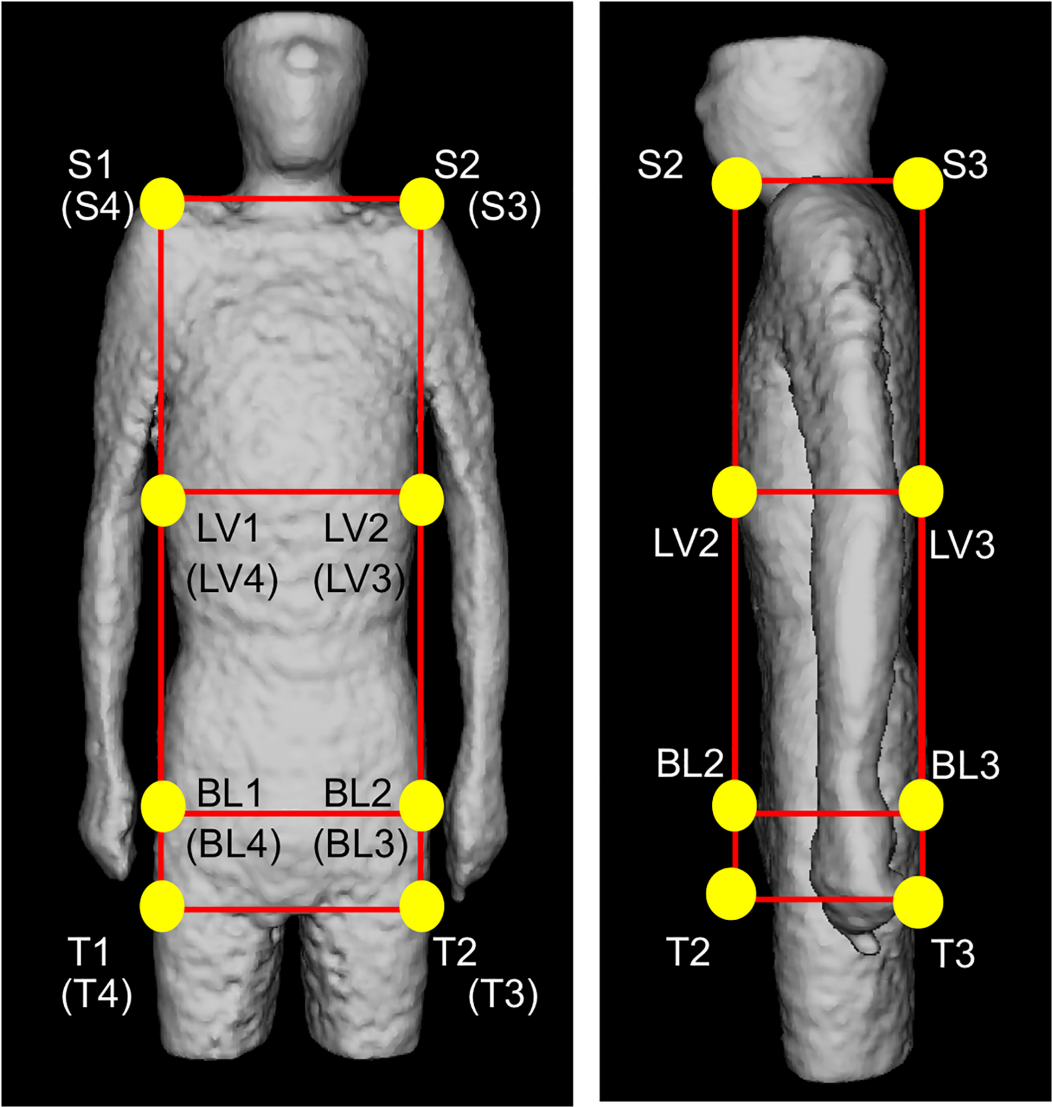
Automated configured points to represent NS, TH, A, P, BL and LV planes.

Physique registration was performed by using an affine transformation after the whole torso area was extracted from the scan volume. Fig 1 shows the 6 planes used for determining the torso region. The neck-shoulder (NS) plane was determined by measuring the area of the body in each axial plane. The plane showing a minimum area was selected as the NS plane [Fig 1 (A)]. The arms-chest (AC) planes were identified by locating the axilla [Fig 1 (B)]. The axilla positions were identified on the graph showing the total count of body area in the sagittal plane vs sagittal body positions. Fig 2 shows an example of the area distributions vs slice index in the axial and sagittal scans for determining the NS and AC planes, respectively. The thigh-hips (TH) plane [Fig 1(C)] was determined using a rule-based technique for analyzing the body region labels in each axial scan. Fig 3 shows the labeled images of the hip/waist and thigh regions. The thigh regions were identified as 2 large circular areas in an axial image, and they united at the base of the hip/waist region. The TH plane was defined as the first plane where the thigh areas united when assessing the axial planes in the tail-to-head direction. The anterior and posterior (A, P) planes were determined by locating the front and back surfaces in body space. To determine the A and P planes, we plotted the total count of body area in each coronal plane (Fig 4). The beginning and end of the distribution define the A and P planes, respectively. The head region was excluded from the calculation of body area in each slice. Using the 6 boundary planes, the torso region was confined in a cuboid. The 4 corners in the NS plane, TH plane, A plane, P plane, left AC plane, and right AC plane were labeled {S1, S2, S3, S4}, {T1, T2, T3, T4}, {S1, S2, T2, T1}, {S4, S3, T3, T4}, {S1, T1, T4, S4}, and {S2, T2, T3, S3}, respectively, as illustrated in Fig 5.

Organ registration was performed by 2 approaches: the first approach was based on the automatic recognition of the bladder and the second was based on the identification of a plane between the liver and the lung. In the first approach, because of the excretion of FDG in the urine, the SUV of the bladder was so high that a thresholding technique could easily extract the bladder region. This technique was applied to each coronal plane to extract the bladder slices. The average of the center of gravity from each bladder label was calculated. The coordinate along the longitudinal axis was used to determine the bladder (BL) plane in the axial view. The BL plane divided the cuboid at 4 points {BL1, BL2, BL3, BL4}, as shown in Fig 5.

The thresholding and labeling technique in the 2D coronal plane can be used to extract the liver after the cuboid was defined. The apex point of the liver to the head was defined as the boundary between the liver and lung, and the axial coordinate was selected as the liver (LV) plane. The LV plane also divided the cuboid at points {LV1, LV2, LV3, LV4}, as shown in Fig 5.

By combining the 16 abovementioned control points, the torso region could be represented by using 8 planes. These 16 automatically extracted points were used to deform the image to match it to the standard body, which had 16 identical control points that were set manually. The affine transform was employed to deform the shapes in the form of a rigid transformation. The nearest neighbor interpolation technique was also used to compensate for the voxel gaps caused by the deformation.

**Fig 6:**
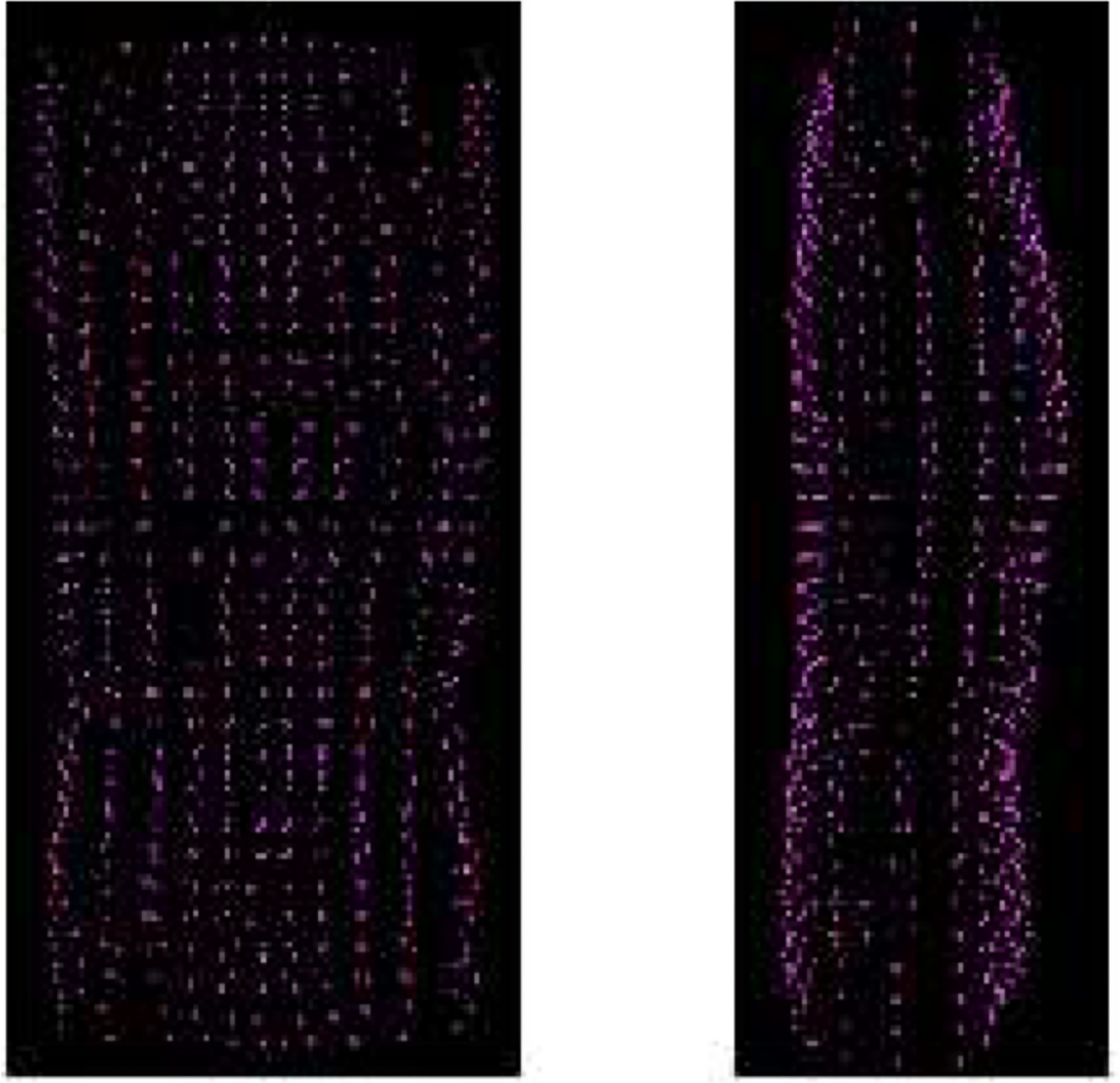
Given control points to register body surface.

Surface registration was performed by using the thin-plate spline (TPS) technique[21]. The TPS has been used as a non-rigid transformation model. The TPS technique involves setting control points on the original and new shapes and deformation of the original shape to the new shape by spatial mapping with smooth interpolation. First, we set the control points on the standard body because the patient’s torso had to be deformed. The same number of control points could also be set on the patient’s body surface (Fig 6). The control points were set on the body surface at 15-degree intervals on each of 5 axial scans (approx. 20 mm). The number of control points was 768 in males and 672 in females.

**Fig 7:**
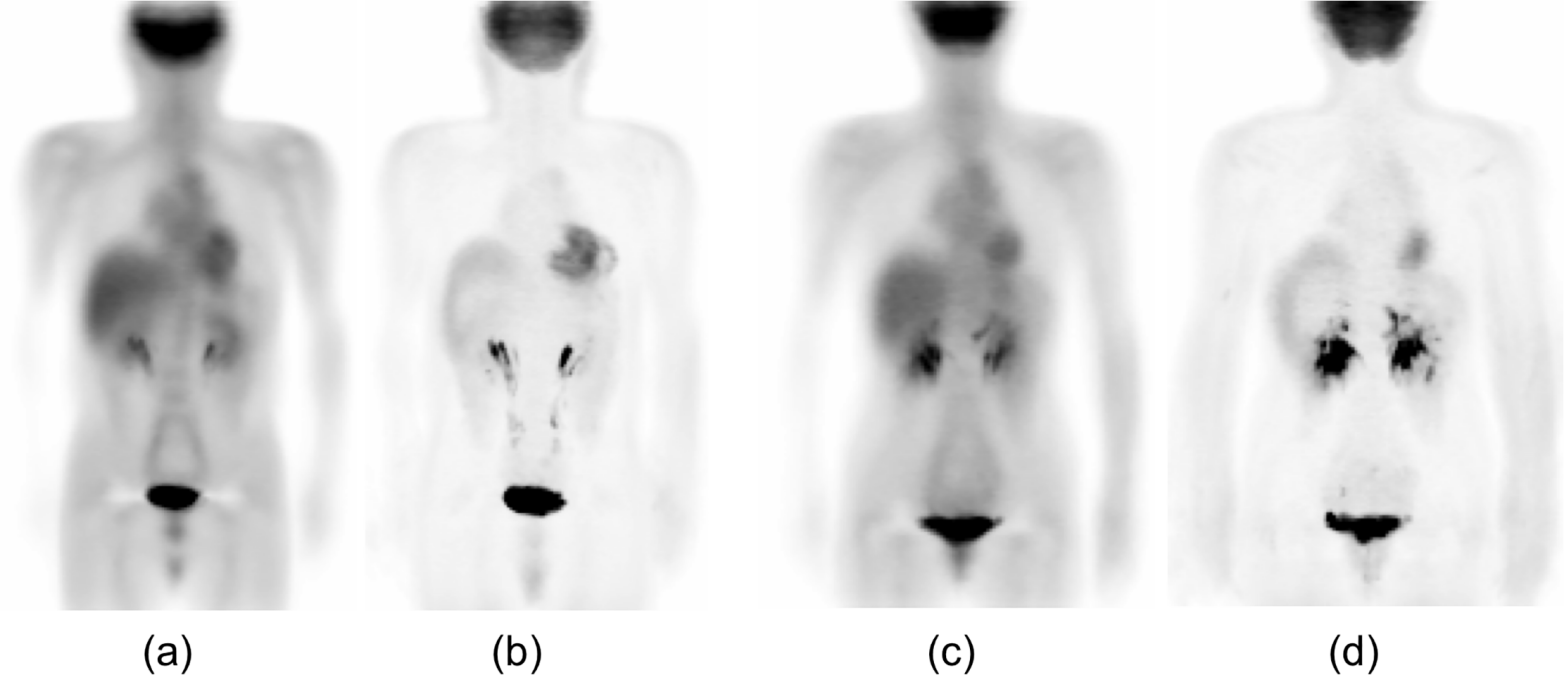
Calculated distributions of mean and SD in SUV for male and female in a coronal plane. Actual distributions are in 3D. (a) Mean distribution for male. (b) SD distribution for male. (c) Mean distribution for female. (d) SD distribution for female.

The mean and SD distributions for males and females were calculated as the registration results of all cases, as shown in Fig 7. We combined the mean and SD distributions in a 3D space as a statistical model of FDG-PET scan images to represent normal metabolism and estimated the confidence interval for every organ in the torso region.

### Z-score

Patient scans were deformed by using the same procedure that was used to configure the statistical models. The SUVs in the patient scans were converted into Z-scores formed on a pixel-by-pixel basis as follows:

$$Z\text{-}score\left(x,y,z\right)=\frac{P\left(x,y,z\right)-M\left(x,y,z\right)}{SD\left(x,y,z\right)}$$ Eq 1

where *M*(x, y, z) and *SD*(x, y, z) represent the mean and SD of the normal group after the image deformation, respectively. *P*(x, y, z) represents the SUV after the scans were deformed to the predefined torso configuration.

## Results

**Fig 8:**
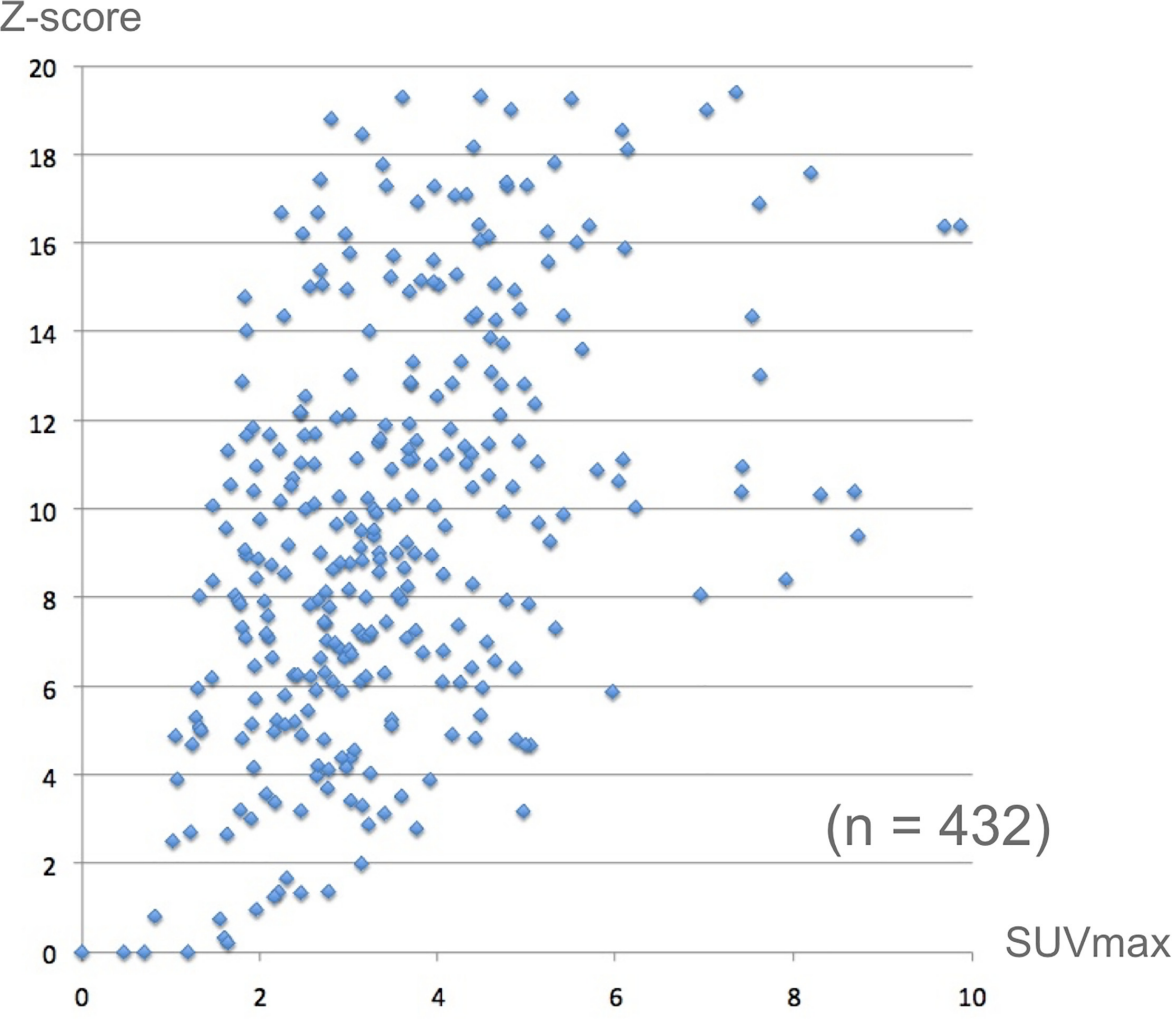
Distribution of SUVmax vs Z-score. It is apparent that the Z-scores of the most abnormal cases are greater than 2.0.

The Z-scores of 417 out of 432 abnormal spots were larger than 2.0, which indicates the statistical abnormalities, as shown in Fig 8. The Z-scores of 6 out of 250 suspicious spots, in which the SUVmax was between 2.0 and 5.0, were less than 2.0. A higher Z-score indicates increased metabolic activity with high severity, but the suspicious accumulations between 2.0 and 5.0 in SUVmax tend to have high Z-scores.

**Fig 9:**
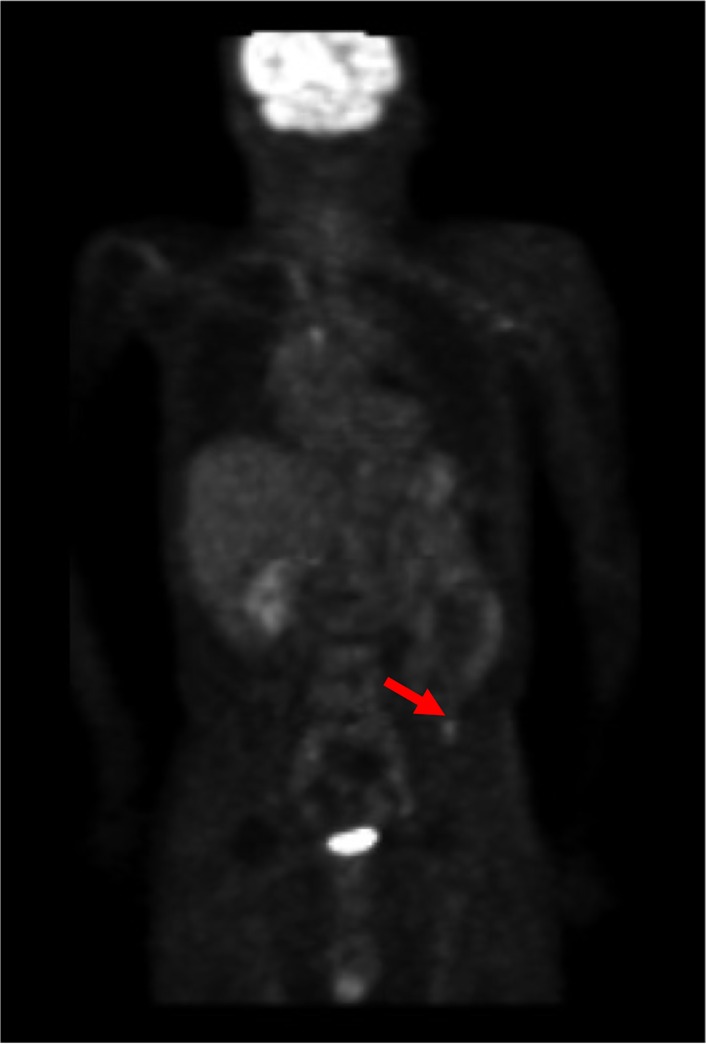
Example of abnormal accumulation (colon cancer). The SUV of 2.1 was converted into Z-score of 5.73, because the mean and SD at the location (an arrow) were 0.61 and 0.26, respectively.

**Fig 10:**
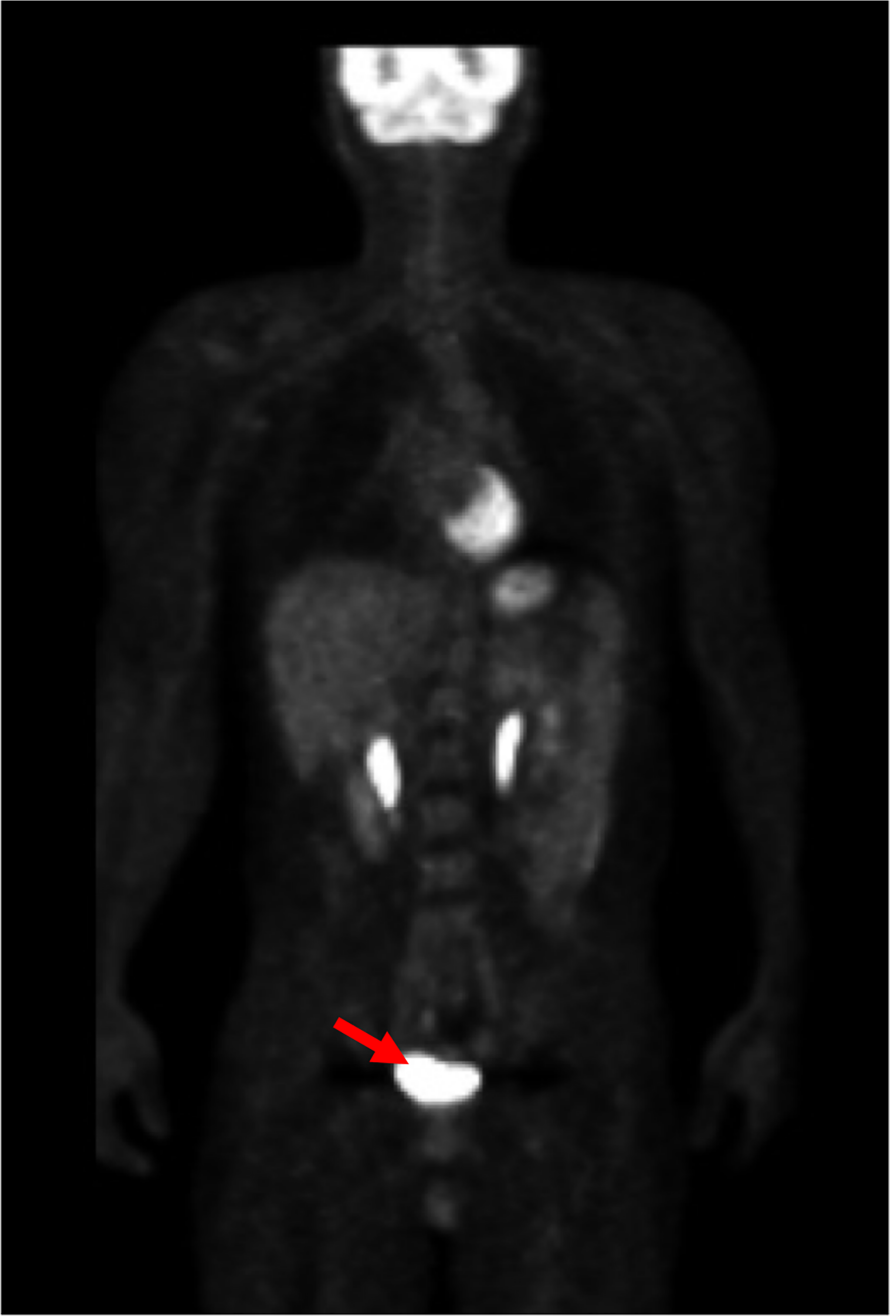
Example of normal accumulation (bladder). The SUV of 65.4 was converted into Z-score of 1.84, because the mean and SD at the location (an arrow) were 19.6 and 24.9, respectively.

The SUV of the spot as shown in colon cancer (Fig 9) was 2.1. The mean and SD at the location (an arrow) were 0.61 and 0.26, respectively. The SUV could be converted into 5.73 by using the statistical methods (Eq 1). A normal accumulation in the bladder was converted into a normal Z-score. The very high accumulation, such as 65.4 in SUV, was converted into 1.84 in Z-score (Fig 10).

## Discussions

**Fig 11:**
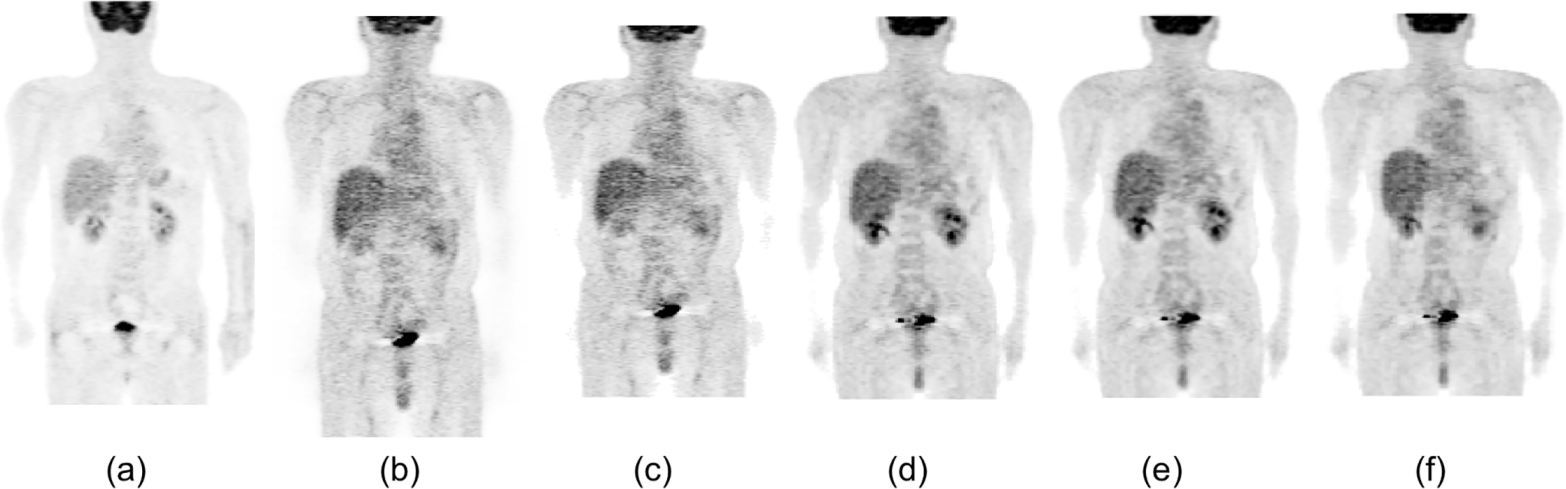
Standard body and registration example in a coronal plane. (a) A male standard body selected from normal database. (b) An another normal body to be deformed to the standard body shown in (a). (c) A result of physique registration. (d) A result of physique and bladder registration. (e) A result of physique, bladder, and liver registration. (f) A result of physique, organ, and surface registration.

We used our semi-automated approach based on a 3D image deformation technique to obtain the statistical model used for analyzing the scan images. Although the technique reduces the need for complicated procedures to select regions of interest (ROIs) in the 3D volume, the registration result may affect the mean and SD values. Fig 11 shows the effect of the various registration step on the standard and other normal bodies. A comparison of Fig 11(A) with Fig 11(B) showed that the positions of the liver, bladder, and other organs and the physique were quite different from each other. The physique registration result, as shown in Fig 11(C), approximately matched the standard body, but the position of the bladder was not matched on the standard body. The results of physique and bladder registration are shown in Fig 11(D). The bladder position, NS plane, and TH plane are well registered, but the position of the right lobe of the liver is slightly different. Organ registration for the liver and bladder improved the registration result, as shown in Fig 11(E). Miss-registration often occurs around the body surface; therefore, surface registration should be applied, as shown in Fig 11(F). These results show the effectiveness of each registration step.

As configuring the statistical model, the analysis of SUV showed the variety of normal organs. The SUV ranges of normal organs in the female lung and male liver were 0.52 and 1.73, respectively. This result implies that the SUVs of normal structures range widely depending on the organ and region. The mean SUVs obtained in our study were comparable to those obtained in another important study that analyzed the mean and SD of SUVs in normal organs(9). This study reported that the mean ± SD of SUV in the liver were 2.06 ± 0.45 for group-a (age, 30–55 years) and 2.18 ± 0.44 for group-b (age, 56–80 years). We used a t test to compare their results with ours and determined the statistical significance (p < 0.01). This may be caused by differences in the radioactivity of injected FDG. This study used an FDG dose of 540 ± 66 MBq, which was higher than that used in our study (140–200 MBq).

Statistical analysis showed that the SUVs of abnormal accumulations and normal accumulations were significantly different. The mean SUVs ranged from 4.81 to 6.51. In addition to the ranges of normal patterns and these abnormal accumulations, the familiarity with the normal and abnormal patterns may improve the diagnostic accuracy not only for the detection of cancers but also for the estimation of chemotherapy doses for cancer treatment. The familiarity by using our developed scheme was obtained as a normal model using the mean and SD in computer software because manual procedures in the SPM and 3D-SSP were completely excluded.

The proposed technique for deformation of the torso region may enable temporal subtraction between two time-series scans of pre- and post-chemotherapy or previous and current examinations to enhance the differences of SUVs. Shiraishi et al. proposed a bone subtraction technique for 2D images, and this technique showed a good performance in observer studies for detecting metastasis[22]. Kano et al. also proposed the fundamental deformation technique in 2D chest images[23]. In our proposed method, the subtraction between the previous and current scans can be easily performed because the torso regions have the same shape. Interpretations of the Z-scores’ differences can also be achieved by using our developed statistical analysis method.

The organ segmentation of CT images from PET/CT scan will be the next challenge for the complete anatomical standardization approach to constructing the normal models. We have also been developing an automated organ recognition method for CT images [24, 25]. Despite of low image quality of CT images from PET/CT device, we suppose that the organ locations can be recognized with an accuracy around 5-10mm of PET image resolutions in our primary study. The combination of organ recognition and the idea of deformation proposed in this study will cover the fully automated anatomical standardization with high accuracy of organ registrations.

## Conclusions

We have developed an automated image registration scheme for FDG-PET. Even if the simple image registration scheme was applied to configure the statistical model of SUV in the torso region as the anatomical standardization approach, our computerized scheme would be useful for visualization and detection of subtle lesions on FDG-PET scan images. Further, the computerized scheme can be used even when the SUV may not show an abnormality clearly as a Z-score. This technique may be applied in computer-aided diagnosis/detection software used for analyzing FDG-PET scan images.
